# Multi-Core Time-Triggered OCBP-Based Scheduling for Mixed Criticality Periodic Task Systems

**DOI:** 10.3390/s23041960

**Published:** 2023-02-09

**Authors:** Marian D. Baciu, Eugenia A. Capota, Cristina S. Stângaciu, Daniel-Ioan Curiac, Mihai V. Micea

**Affiliations:** 1 Computer and Information Technology Department, Politehnica University Timisoara, V. Parvan 2, 300223 Timisoara, Romania; 2 Automation and Applied Informatics Department, Politehnica University Timisoara, V. Parvan 2, 300223 Timisoara, Romania

**Keywords:** mixed criticality systems, real-time scheduling, embedded systems, non-preemptive scheduling, multiprocessor systems, time-triggered scheduling

## Abstract

Mixed criticality systems are one of the relatively new directions of development for the classical real-time systems. As the real-time embedded systems become more and more complex, incorporating different tasks with different criticality levels, the continuous development of mixed criticality systems is only natural. These systems have practically entered every field where embedded systems are present: avionics, automotive, medical systems, wearable devices, home automation, industry and even the Internet of Things. While scheduling techniques have already been proposed in the literature for different types of mixed criticality systems, the number of papers addressing multiprocessor platforms running in a time-triggered mixed criticality environment is relatively low. These algorithms are easier to certify due to their complete determinism and isolation between components of different criticalities. Our research has centered on the problem of real-time scheduling on multiprocessor platforms for periodic tasks in a time-triggered mixed criticality environment. A partitioned, non-preemptive, table-driven scheduling algorithm was proposed, called Partitioned Time-Triggered Own Criticality Based Priority, based on a uniprocessor mixed criticality method. Furthermore, an analysis of the scheduling algorithm is provided in terms of success ratio by comparing it against an event-driven and a time-triggered method.

## 1. Introduction

Embedded real-time systems are becoming more present in our everyday life, from fields such as automotive, avionics, military and industrial control systems to medical equipment and even domestic applications and Internet of Things. A new trend in the design of real-time and embedded systems is the integration of components with different criticality levels into the same hardware platform. Mixed criticality systems (MCSs) are “embedded computing platforms in which application functions of different criticality share computation and/or communication resources” [1]. Additionally, these platforms are migrating from single cores to multi-cores due to an increase in application complexity and strict requirements such as cost, space, weight, power consumption and so on.

While multiple scheduling techniques have already been proposed in the literature for different types of mixed criticality systems, the number of papers addressing multiprocessor platforms running in a time-triggered mixed criticality environment is relatively low compared to event-driven approaches [2,3].

For a time-triggered environment, activities in the system are triggered by the progression of time [4]. The scheduling decisions made at each time instant follow the pre-computed schedule stored in a scheduling table. These scheduling tables offer simplicity, isolation between components of different criticalities, determinism and are easy to verify, thus, they are very popular in safety critical systems subject to certification (ex. industrial applications) [5]. In a mixed criticality platform, scheduling tables are constructed for each criticality level of the system.

This current paper is an extended version of the paper [6] presented at the International Symposium on Electronics and Telecommunications (ISETC) 2022, which proposed a non-preemptive, table-driven algorithm for scheduling periodic tasks on a multiprocessor platform. The method is based on the uniprocessor table-driven algorithm presented by Baruah et al. [5], which uses the Own Criticality Based Priority technique for constructing the job priority list. The main contributions of this paper [6] are listed below:

- The extension of a mixed criticality uniprocessor table-driven scheduling algorithm to a mixed criticality algorithm for periodic tasks on a multiprocessor platform (Section 4.1, Section 4.2 and Section 4.6). The original method has been modified to employ a periodic mixed criticality job model (Section 4.4 and Section 4.5).
- The proposal of a task partitioning heuristic for the multiprocessor mixed criticality system (Section 4.3).
- The comparison of the newly developed algorithm in terms of success ratio with two state-of-the-art methods (Section 5).
- This current paper aims to demonstrate the efficiency of algorithm [6] through:
- More experiments and comparisons.
- Additional details about the algorithm implementation.

The remainder of this paper is structured as follows: Section 2 covers some related work regarding event-driven and time-triggered scheduling algorithms in mixed criticality systems. Section 3 addresses the scheduling problems of the time-triggered real-time mixed criticality systems. In Section 4, the Partitioned Time-Triggered Own Criticality Based Priority (P-TT-OCBP) multiprocessor scheduling algorithm is introduced and explained, while in Section 5 we analyze the performance of our method by comparing it against an event-driven scheduling method (P-EDF-VD) and a time-triggered algorithm (P_FENP_MC) in terms of success ratio. Finally, Section 6 summarizes the conclusions.

## 2. Related Work

Since Vestal’s initial work [7] a number of studies have been introduced for mixed criticality scheduling. Algorithms in mixed criticality systems (MCSs) can be classified based on their scheduling points (i.e., the moments in time when scheduling decisions occur) into two main categories: event-driven and time-triggered.

### 2.1. Event-Driven Scheduling Algorithms

Research in real-time scheduling for MCSs has been centered around event-driven approaches. In event-driven scheduling, the scheduling points are defined by task completion and task arrival events [8]. Some examples of event-driven schedulers are [9,10,11,12,13].

A well-known event-driven scheduling algorithm in MCSs is Earliest Deadline First with Virtual Deadlines (EDF-VD) [9] for two criticality levels (Hi (high) criticality and Lo (low) criticality). Under EDF-VD, if the system is in Lo mode, each high criticality task is assigned a virtual deadline, which is earlier than its actual deadline. If the system is in Hi mode, high criticality tasks are scheduled according to their real deadlines.

By extending EDF-VD to support adaptive task dropping under task-level mode switch, two uniprocessor algorithms were introduced in [10], namely EDF-Adaptive task Dropping and EDF-AD-E (Enhanced). For multiprocessor platforms, a method is described in [11] based on setting virtual deadlines from any feasible fluid rates, while in [12], a fluid-based algorithm was implemented, which allows tasks to execute on the same processor simultaneously. In [13], a semi-partitioned mixed criticality method is presented, which offers low criticality task migration from one processor to another once mode switch occurs, in order to improve the service of low criticality tasks in the high criticality mode.

### 2.2. Time-Triggered Scheduling Algorithms

Despite the popularity of event-driven algorithms, current practice in many safety critical application domains favors time-triggered (TT) methods due to their complete determinism, which facilitates certification. In the TT paradigm, scheduling decisions are made at predetermined points in time [4]. Thus, a schedule is computed prior to run-time for the entire execution of the system and is represented in a scheduling table. Each scheduling decision made during run-time is determined by examining this scheduling table. To the best of our knowledge, few papers have addressed time-triggered scheduling in MCSs [4,14,15,16,17].

A scheduling algorithm with real-time, non-preemptive and table-driven characteristics for MCSs is proposed in [14]. This algorithm guarantees a perfectly periodical execution in time-triggered mixed criticality environments and was implemented in two variants, i.e., Fixed Execution Non-Preemptive Mixed Criticality (FENP_MC) and P_FENP_MC (Partitioned), to meet the demands of both uniprocessor and homogeneous multiprocessor settings. The main advantage is that the algorithm assures a perfectly periodical (i.e., jitter-less) task execution in time-triggered mixed criticality environment, but its main disadvantage is that it has a relatively low success ratio for a high processor utilization of the task set to be scheduled.

The time-triggered algorithm presented in [15] is specifically designed for uniprocessor platforms and applies dynamic voltage and frequency scaling to reduce the energy consumption. This paper proposes the first energy-efficient time-triggered algorithm for MCSs. The schedule constructed by energy-efficient TT-Merge outperforms energy-efficient EDF-VD [18]. However, the algorithm uses continuous frequency levels; therefore, it might not be optimal with respect to energy consumption for discrete frequency levels, which are more common in practice. Another noteworthy algorithm, but this time explicitly developed for identical multiprocessor platforms executing mixed criticality tasks, is reported in [16]. This algorithm performs better than previous time-triggered, multiprocessor methods [17] in terms of scheduling overhead.

Baruah et al. [5] offer a method for building scheduling tables to allocate jobs’ priorities according to the Own Criticality Based Priority (OCBP) algorithm [19]. By this, a correct scheduling strategy driven by a priority-based mechanism has been provided. Our algorithm extends this approach for building scheduling tables with the specific case of periodic tasks, by considering the periodic task set as a collection of independent jobs which explicitly enumerates all the jobs in the system. This paper also provides a partitioning heuristic for the case of multiprocessor platforms. To our knowledge, very few time-triggered algorithms for multiprocessor platforms exist in the literature.

Our time-triggered algorithm offers complete determinism and isolation between components of different criticalities in comparison to the event-driven multiprocessor algorithms described previously [11,12,13]. This ensures the certification of high criticality functionalities under very conservative assumptions. In general, isolation between components of different criticalities can cause very low resource utilization. This happens because platform resources are reserved for the exclusive use of high criticality functionalities in order to meet certification requirements under pessimistic assumptions. Due to isolation, these resources cannot be reclaimed by less critical applications. However, the uniprocessor time-triggered algorithm which our paper extends allows high utilization of platform resources under less pessimistic assumptions. We have also decided to adapt the algorithm for periodic tasks because they are independent, run cyclically and their characteristics are known in advance.

In the following sections, the proposed scheduling algorithm is described, analyzed and compared in terms of success ratio to two multiprocessor methods from the literature, an event-driven and a time-triggered technique.

## 3. Model and Problem Statement

The problem which we address in this paper is to implement a multiprocessor mixed criticality scheduling algorithm by adapting a classical algorithm [5] to a periodic task execution model and also to extend it from a uniprocessor system to a multiprocessor one [20].

In this section, we formally define the mixed criticality job model used. For a dual criticality system, we used a task model with the following properties, based on the standard MCSs model [7,21] and an extension for periodic tasks [14]:

- An MCS executes in either of two modes: Hi-criticality mode or Lo-criticality mode.
- Each mixed-criticality task *τ_i_* is characterized by a set of parameters [7,14]:
  (1) $$\tau_{i}=\left\{\begin{array}{c} T_{i},\;D_{i},\;L_{i},\;\left\{C_{i,\;L_{j}}\;|\;j\in \left\{Lo,\;Hi\right\}\right\},\;\\ \left\{S_{i,\;L_{j}}\;|\;j\in \left\{Lo,\;Hi\right\}\right\} \end{array}\right\}$$
  where *T_i_*, *D_i_* and *L_i_* denote, respectively, the period, the deadline and the criticality level (i.e., *Lo* or *Hi*) of the task *i*; *C_i,Lj_* is a vector containing the worst-case execution times (WCETs) for each criticality level; and *S_i,Lj_* is a vector where each element represents the execution start time, relative to its release time, for each criticality level that is lower than or equal to the task criticality level *L_i_*, with *S_i,Lj_ < D_i_*.

- A task consists of a series of jobs that inherit some of the parameters of the task (*T_i_*,*D_i_*,*L_i_*). Furthermore, each job adds its own parameters, which means that the *k*-th job of task *i* is characterized by the following:
  (2) $$J_{i,\;k}=\left\{a_{i,\;k},\;d_{i,\;k},c_{i,\;k},\;s_{i,\;k},T_{i},\;D_{i},\;L_{i}\right\}$$
  where:
  ○ *a_i,k_* represents the arrival time of job *k*, with *a_i,k+1_* − *a_i,k_* ≥ *T_i_*.
  ○ *d_i,k_* is the absolute deadline of job *k* and can be obtained using *d_i,k_* = *a_i,k_* + *D_i_*.
  ○ *c_i,k_* expresses the execution time and depends on the criticality mode of the system (e.g., for *L* = *L_o_*, *c_i,k_* = *C_i,Lo_*).
  ○ *s_i,k_* offers the absolute execution start time corresponding to job *k* and, similar to *c_i,k_*, also depends on the criticality mode of the system.

## 4. Algorithm P-TT-OCBP

This section describes the mapping heuristic used for partitioning tasks to processors and the non-preemptive scheduling algorithm implemented at the processor level. As mentioned before, the algorithm is an extension of the method described by Baruah et al. in [5].

### 4.1. Original Algorithm 

The original algorithm [5] uses a sufficient MC-schedulability test, namely the Own Criticality Based Priority [19] to find a complete ordering of the jobs. The priority assignment list is constructed offline (Algorithm 1).
**Algorithm 1:** Own Criticality Based Priority.**Input:**$\Delta_{p}$ (the job list for processor p)**Output:** $\Upsilon_{p}$ (the priority list for processor p)sort $\Delta_{p}$ in non-decreasing order by $d_{i,\;k}$
**for** $k\in \left\{0,\;1,\;\ldots ,\;size\;of\;\Delta_{p}\right\}$ **do**

        sumLo←0
        sumHi←0
        **for**
$j\in \left\{0,\;1,\;\ldots ,\;size\;of\;\Delta_{p}\right\}$ **do**
                   **if** j≠k **then**
                            $sumLo\leftarrow sumLo+c_{j,\;Lo}$
                            **if** $L_{k}=Hi$ **then**
                                     $sumHi\leftarrow sumHi+c_{j,\;Hi}$
                            **end if**
                    **end if**
        **end for**
        **if** $L_{k}=Lo$ **and**
$d_{k}-sumLo\geq c_{k,\;Lo}$ **then**

                            add $J_{k}$ to $\Upsilon_{p}$
       
**end if**
        **if** $L_{k}=Hi$ **and**
$d_{k}-sumLo\geq c_{k,\;Lo}$ **and**
$d_{k}-sumHi\geq c_{k,\;Hi}$ **then**

                             add $J_{k}$ to $\Upsilon_{p}$

        **end if**
**end for**

The job with the lowest priority is determined first: the lowest priority may be assigned to a job *J_k_* if there is at least *c_k,Lj_* units of time between its arrival time and its absolute deadline available when every other job *J_x_* is executed before *J_k_* for *c_x,Lj_* units of time. OCBP assumes that every job, other than *J_k_*, has priority over *J_k_* and ignores whether these jobs meet their deadlines or not. The algorithm is applied repeatedly to the set of jobs (excluding the lowest priority job), until all the jobs are ordered, or at some iteration, a lowest priority job does not exist [22,23].

In [22], the OCBP method was compared, in terms of processor speedup factor, to two techniques used for resource allocation and scheduling in MCSs and it was concluded that the OCBP-schedulability test has better performance.

### 4.2. Working Hypothesis

For the proposed scheduling algorithm, we have considered a homogenous multicore (where the number of cores is equivalent to the number of processors), non-preemptive, dual criticality system (i.e., a mixed criticality system with two criticality levels: low and high), running periodical tasks.

- A dual criticality system is defined to execute in one of two modes: Lo-criticality mode and Hi-criticality mode.
- Each job is characterized by the set of parameters described in (2), with C(Lo) ≤ C(Hi).
- The system starts in Lo-criticality mode and does not change as long as jobs execute within their Lo-criticality WCETs.
- If any job overruns its Lo-criticality WCET, then a criticality mode change occurs.
- As the system instantly moves to Hi-criticality mode, all Lo-criticality jobs are dropped (they are no longer executed). Hi-criticality jobs are allowed to run according to their Hi-criticality WCETs.
- The system remains in Hi-criticality mode.
- In this paper, we only consider the mode change from Lo-criticality to Hi-criticality.

### 4.3. Partitioning Tasks to Processors

As the demand for increased performance and general-purpose programmability grows, general-purpose multi-core processors are being adopted in all segments of the industry. By adding more specifications while preserving reasonable power characteristics, parallel processing improves performance [24]. Thus, our algorithm was developed for mixed criticality multiprocessor platforms.

The task mapping algorithm that we are using is based on a well-known task partitioning heuristic from the literature, namely first fit decreasing (FFD) [25].

Tasks are selected one by one from the task set and added in each processor, where two conditions must be verified: the current processor utilization (for both Lo-criticality mode and Hi-criticality mode), which is the sum of utilizations for all the tasks on the processor, must not exceed 1 [26]. Tasks are sorted in non-decreasing order of their periods. 

The task partitioning method is described below:

- The utilization of each task is computed based on the criticality level (3): for Hi-criticality tasks there will be two utilizations (one for each criticality level).

(3) $$U_{i,L_{j}}=\frac{C_{i,\;L_{j}}}{T_{i}}$$

- Tasks are selected one by one from the task set and added into each processor where a test is performed.
- Two conditions must be verified (4):

(1) The current total processor utilization in Lo-criticality mode $U_{P_{q}}\left(Lo\right)$ must not exceed 1.

(2) The current total processor utilization in Hi-criticality mode $U_{P_{q}}\left(Hi\right)$ must not exceed 1.

(4) $$U_{P_{q}}\left(Lo\right)\leq 1\;and\;U_{P_{q}}\left(Hi\right)\leq 1$$

- If the above two conditions are met, the task will be assigned to *P_q_* and the total processor utilizations are updated.
- If one of the two conditions returns failure, the task is removed from *P_q_* and added into the next processor, where the same test is performed

These steps are repeated until all the tasks are partitioned into processors.

### 4.4. Constructing the List of Jobs at the Processor Level

The periodic tasks on each processor are represented as a collection of independent jobs, obtained by explicitly enumerating all the jobs over the hyperperiod interval.

Each job inherits a set of parameters from the task (*T_i_*,*D_i_*,*L_i_*,*C_i_*), to which we add an arrival time and absolute deadline of the job according to (5) and (6):

(5) $$a_{i,\;k}=a_{i,k-1}+T_{i}$$

(6) $$d_{i,\;k}=a_{i,\;k}+D_{i}$$

### 4.5. Scheduling at the Processor Level

The priority list is constructed using an algorithm called Own Criticality Based Priority (OCBP) [5], where priorities are assigned to jobs based on the following criteria:

- The job list to be prioritized must be parsed in non-decreasing order of deadlines *d_i,k_*.
- The criticality level of the first job k from the list is verified:
  ○ If the criticality level is Lo we compute the sum of the Lo-criticality WCETs (*sum(Lo)*) for the rest of the jobs.
  ○ If the criticality level is Hi we compute two sums, one for Lo-criticality WCETs (*sum(Lo)*) and one for Hi-criticality WCETs (*sum(Hi)*) for the rest of the jobs.
- Next, the algorithm checks if job k can be added in the priority list, depending on its criticality level:
  ○ For a Lo-criticality level:
    (7) $$d_{i,\;k\;\;}-sum\left(Lo\right)\geq C_{i,\;L_{Lo}}$$
  ○ For a Hi-criticality level, two conditions must be met:

(8) $$\{\begin{array}{c} d_{i,\;k\;}-sum\left(Lo\right)\geq C_{i,\;L_{Lo}}\\ d_{i,\;k}-sum\left(Hi\right)\geq C_{i,\;L_{Hi}} \end{array}$$

- If these conditions are met, job *k* is moved from the list of jobs to the priority list. Otherwise, the next job *k* + 1 in the list is taken, until the entire list of jobs is verified.
- If jobs are still in the list after the list of jobs is parsed at least once, the same algorithm is computed again, until no more jobs are left.
- If at least two jobs remain in the list of jobs which cannot be prioritized, the set of tasks is deemed not schedulable.
- The resulting priority list is sorted in non-decreasing order of deadlines. The schedule is constructed based on the priority list as follows:
- The first job is extracted from the priority list, with *s_i,k_* = 0.
- We then compute the completion time (ct_i,k_) of the job:

(9) $$ct_{i,\;k}=s_{i,\;k}+c_{i,\;k}$$

- For the next *k* − 1 jobs, we compare the arrival time with the previous job completion time: if the completion time is greater than the arrival time, then the start time will take on the value of the previous job completion time; otherwise, the start time will be equal to the current job arrival time. The completion time is computed using Equation (9).

Our scheduler creates, in an offline phase, two dispatch tables for each processor (one for the Lo-criticality mode and one for the Hi-criticality mode), called scheduling tables.

The scheduling table for processor q (ȴ*_q_*) is presented below as an array of structures:

(10) $${ȴ}_{q}=\left\{TaskID,JobID,StartTime\right\}$$

where ȴ*_q_* is sorted in non-decreasing order of the job start time on each processor.

Next, we present an example in order to illustrate the construction of our scheduling tables for a single processor platform. Let us consider the task set presented in Table 1:

Table 2 and Table 3 illustrate the scheduling tables for Lo-criticality mode and Hi-criticality mode of the task set example, presented in Table 1.

### 4.6. System Execution

**Figure 1:**
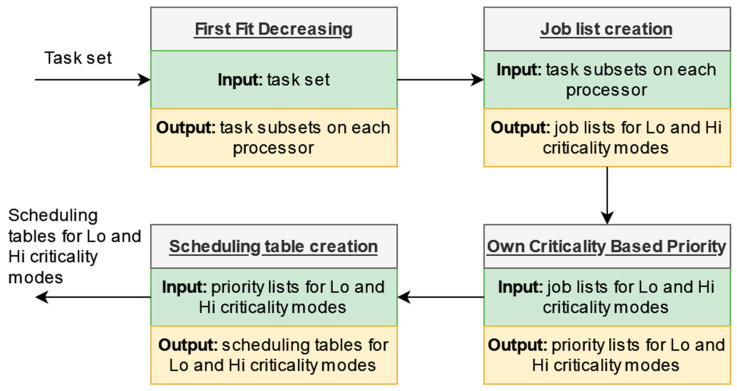
System execution flowchart.

The system execution flowchart is illustrated in Figure 1. Each task in the task set is partitioned on processors using the first fit decreasing (FFD) algorithm [25]. At the processor level, two job lists are created (a list for all the jobs on the processor and a list containing only the jobs of Hi-criticality tasks) by explicitly enumerating the jobs of the tasks assigned to the processor. Then, the priority list construction is verified by using the Own Criticality Based Priority (OCBP) method for the two modes of the system. If the priority list creation fails, the task set is unschedulable. Otherwise, two scheduling tables are constructed, one for each criticality mode of the system.

## 5. Evaluation

In this section we will undertake an experimental comparison between P-TT-OCBP and two other multiprocessor scheduling methods: an event-driven, non-preemptive algorithm which uses the FFD [25] heuristic for task partitioning to processors, namely Partitioned Earliest Deadline First with Virtual Deadlines (P-EDF-VD) [27] and a table-driven, non-preemptive, perfectly periodical scheduling method, called Partitioned Fixed Execution Non-Preemptive Mixed Criticality (P_FENP_MC) [14]. All tasks were randomly generated in Matlab (R2018b) and the simulation environment was developed for multiprocessor mixed criticality systems, using C++.

### 5.1. Task Set Generation

In our experiments, we employed randomly generated task sets inside a dual criticality platform (Lo, Hi) that were generated using a variant [28] of the workload-generation algorithm provided by Guan et al. [29]. The methodology used for generating the task sets is similar with the one used in [14] and generates the parameters of each new task *τ_i_* as follows:

- Period: *T_i_* is drawn using a uniform distribution on [10, 50].
- Deadline: *D_i_* = *T_i_*.
- Criticality level: *L_i_* = *Hi* with a given probability *P_Hi_*; otherwise, *L_i_* = *Lo*.
- Utilization: *U_i,Lj_* (see Equation (3)), is a vector of size l, where l is the number of criticality levels. The utilizations are generated using five input parameters [28]:
  ○ *U_bound_*:
    (11) $$U_{bound}=max\left(U_{Lo}\left(\tau \right),\;U_{Hi}\left(\tau \right)\right)$$
    (12) $$U_{Lo}\left(\tau \right)=\underset{\tau_{i}\in \tau \;}{\sum }U_{i,L_{Lo}}$$
    (13) $$U_{Hi}\left(\tau \right)=\underset{M_{i}\in Hi\left(\tau \right)}{\sum }U_{i,L_{Hi}}$$
    where *Hi(τ)* is the subset of the entire task set *τ* which contains only the Hi-criticality tasks.
  ○ [*U_L_*,*U_U_*]: The range of the task utilization, with 0 ≤ *U_L_* ≤ *U_U_* ≤ 1.
  ○ [*Z_L_*,*Z_U_*]: The range of the ratio between *Hi*-criticality utilization of a task and *Lo*-criticality utilization, where 0 ≤ *Z_L_* ≤ *Z_U_*.

- WCET: (a) for Lo-criticality level: $C_{i,L_{Lo}}=U_{i,L_{Lo}}\cdot T_{i}$ and (b) for Hi-criticality level: $C_{i,L_{Hi}}=U_{i,L_{Hi}}\cdot T_{i}$ if *L_i_* = *Hi*, otherwise $C_{i,L_{Hi}}=C_{i,L_{Lo}}$.

- Start time: $S_{i,L_{Hi}}=S_{i,L_{Lo}}=0$

### 5.2. Execution Example and Comparison

**Figure 2:**
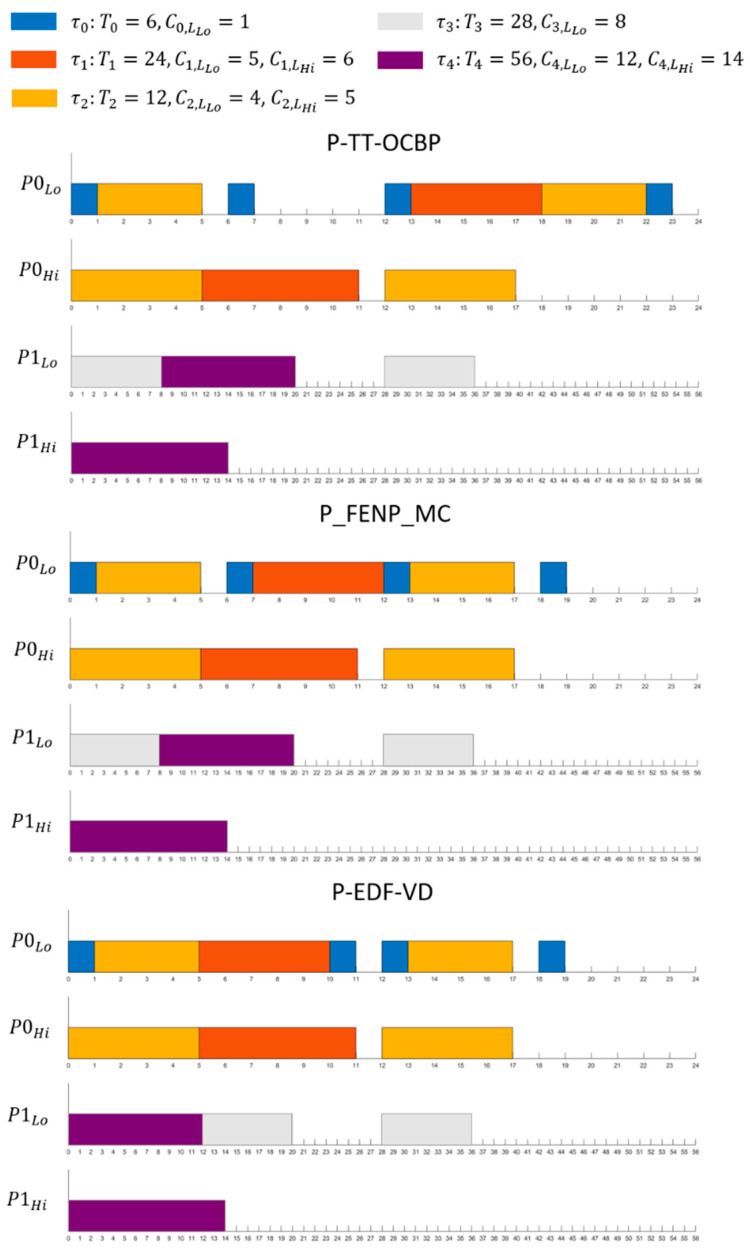
Schedule of the task set example using 3 methods: Partitioned Time-Triggered Own Criticality Based Priority (P-TT-OCBP), Partitioned Fixed Execution Non-Preemptive Mixed Criticality (P_FENP_MC) and Partitioned Earliest Deadline First with Virtual Deadlines (P-EDF-VD), a non-preemptive variant.

An example of a task set is provided in Table 4 in order to illustrate the execution of our scheduling algorithm on a platform with two processors. The same task set is scheduled in Figure 2 using P-EDF-VD and P_FENP_MC for comparison.

Scheduling for both Lo- and Hi-criticality modes is illustrated in Table 4:

### 5.3. Success Ratio

In this section we will undertake an experimental evaluation on a dual criticality, multiprocessor platform between our algorithm P-TT-OCBP and two known scheduling methods in a non-preemptive context: P-EDF-VD and P_FENP_MC.

Each data point from the graph captions is determined by randomly generating 1000 task sets.

**Figure 3:**
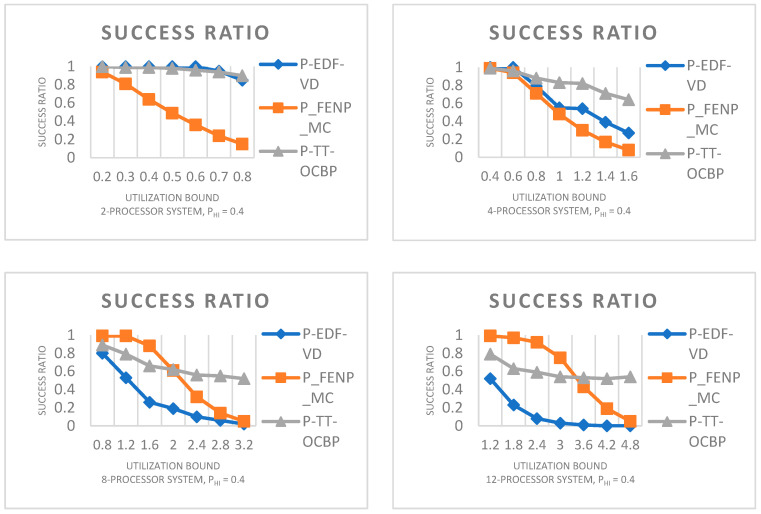
Success ratio by varying the utilization bound. U_L_ = 0.05, U_U_ = 0.75, Z_L_ = 1, Z_U_ = 4.

In Figure 3 we have four graphs with the number of processors ascending from 2 to 4, from 4 to 8 and from 8 to 12 processors. For each graph, the task set utilization bound on the x-axis ranges from 0.2 to 0.8 times the number of processors divided by 2, in steps of 0.1. The results of our experimental evaluation show that our algorithm has a high success ratio in comparison to P-EDF-VD and P_FENP_MC.

**Figure 4:**
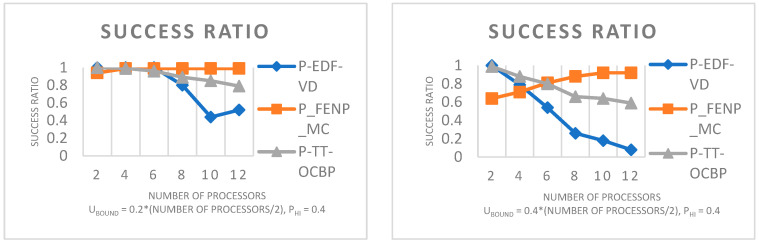

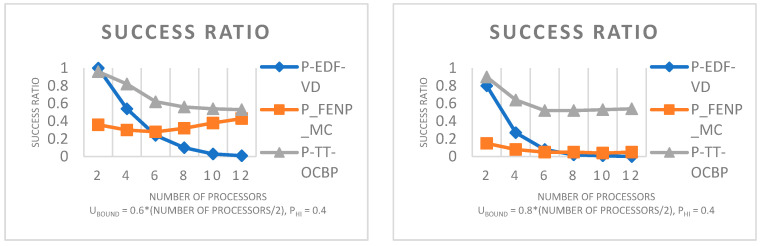
Success ratio by varying the number of processors. U_L_ = 0.05, U_U_ = 0.75, Z_L_ = 1, Z_U_ = 4.

For Figure 4, the number of processors on the x-axis ranges from 2 to 12, in steps of 2. It must be noted that between the four graphs, the Base Utilization bound $\left(U_{bound}=BU_{bound}\times \left(number\;of\;processors/2\right)\right),$ ranges from 0.2 to 0.8, in steps of 0.2. The number of tasks in a task set varies according to the task set utilization bound. Therefore, a lower value on the x-axis decreases the number of tasks in a task set, while a higher value increases it.

An apparently superior performance of P_FENP_MC for the first two graphs can be attributed to the particular implementation of the algorithm, which includes the mapping test executed when partitioning tasks to processors.

**Figure 5:**
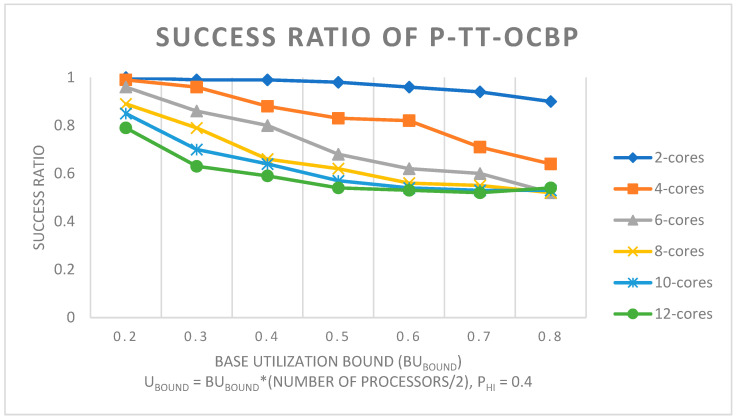
Success ratio of P-TT-OCBP by varying the base utilization bound and the number of processors. U_L_ = 0.05, U_U_ = 0.75, Z_L_ = 1, Z_U_ = 4.

In Figure 5, the task set base utilization bound (BU_bound_) ranges on the x-axis from 0.2 to 0.8, in steps of 0.1. It can be seen that the performance of the algorithm decreases when the number of processors increases. This is because the FFD heuristic, presented in Section 4.3 of this paper, allocates tasks on a processor as long as the total utilization of the processor is lower than or equal to 1. Since the utilization bound is higher when the number of processors increases, $U_{bound}={BU}_{bound}\times \left(number\;of\;processors/2\right),$ there is a higher chance of the task mapping being unsuccessful or the scheduling algorithm at the processor level failing.

## 6. Conclusions

As the complexity of safety critical applications increases, it is important to facilitate certification and to ensure efficient resource utilization. In this paper, we have proposed an algorithm for scheduling periodic tasks on multiprocessor mixed criticality systems, namely Partitioned Time-Triggered Own Criticality Based Priority (P-TT-OCBP). Our approach is based on a polynomial-time algorithm for generating time-triggered schedules and extended to deal with periodic tasks on multiprocessor platforms.

In addition, the algorithm performance was compared with an event-driven method, P-EDF-VD, and a table-driven approach, P_FENP_MC, in a non-preemptive context.

The experimental results show that our algorithm has a high success ratio when the number of processors is low. The higher the number of processors, the lower the success ratio due to the increased total utilization on each processor (the number of tasks scheduled on a processor is determined by the utilization bound). P-TT-OCBP outperforms the other two algorithms in terms of success ratio when the number of processors is low; however, if the number of processors increases, P_FENP_MC performs better due to the additional mapping test executed while partitioning tasks to processors.

As future work, practical implementations of the algorithm can be proposed for different real-time operating systems or real-time extensions of general-purpose operating systems such as Litmus-RT (a multiprocessor RT extension for Linux), which already provides support for time-triggered execution environment. The algorithm can also be adapted to heterogeneous multiprocessor mixed criticality systems.

## Figures and Tables

**Table 1:** Four-task set example.

Task	$T_{i}$	$D_{i}$	$L_{i}$	$C_{i,\;L_{Lo}}$	$C_{i,\;L_{Hi}}$
$\tau_{0}$	8	8	1	4	-
$\tau_{1}$	12	12	2	5	7
$\tau_{2}$	16	16	1	5	-
$\tau_{3}$	24	24	2	1	4

**Table 2:** Lo-criticality mode scheduling table for the task set in Table 1.

TaskID	JobID	StartTime
0	0	0
1	0	4
2	0	5
0	1	10
3	0	14
1	1	15
0	2	16
2	1	20
0	3	25
1	2	29
0	4	32
3	1	36
2	2	37
1	3	42
0	5	43

**Table 3:** Hi-criticality mode scheduling table for the task set in Table 1.

TaskID	JobID	StartTime
1	0	0
3	0	3
1	1	12
1	2	24
3	1	27
1	3	36

**Table 4:** Five-task set example.

Task	$T_{i}$	$D_{i}$	$L_{i}$	$C_{i,\;L_{Lo}}$	$C_{i,\;L_{Hi}}$
$\tau_{0}$	6	6	1	1	-
$\tau_{1}$	24	24	2	5	6
$\tau_{2}$	12	12	2	4	5
$\tau_{3}$	28	28	1	8	-
$\tau_{4}$	56	56	2	12	14
